# Acute coronary syndromes occurring while driving: frequency and patient characteristics

**DOI:** 10.1186/s12199-017-0689-5

**Published:** 2017-12-20

**Authors:** Joji Inamasu, Satoru Miyatake, Takashi Yagi, Shigetaka Noma

**Affiliations:** 1 Department of Emergency Medicine, Saiseikai Utsunomiya Hospital, 1-98 Takebayashi, Utsunomiya, 321-0974 Japan; 2 Department of Cardiology, Saiseikai Utsunomiya Hospital, Utsunomiya, Japan

**Keywords:** Acute coronary syndrome, Driving, Risk factors, Smoking

## Abstract

**Background:**

Acute coronary syndrome (ACS) may occur during any human activity, including driving. The objectives of this study were to report the frequency of ACS occurring while driving, clarify patient characteristics, and analyze the behavioral patterns of drivers who sustained ACS.

**Methods:**

A single-center, retrospective observational study was conducted using prospectively acquired data. Among 1605 ACS patients admitted between January 2011 and December 2016, 65 (60 men/5 women) patients who sustained ACS while driving were identified. Clinical variables were compared between these 65 patients and 1540 patients who sustained ACS while performing other activities. Furthermore, multivariable regression analysis was performed to identify variables associated with ACS.

**Results:**

The frequency of ACS occurring while driving was 4.0% (65/1605). Compared with patients who sustained ACS while performing other activities, those who sustained ACS while driving were significantly younger (66.2 ± 13.0 vs. 57.5 ± 12.2 years, *p* < 0.001) and more likely to smoke (34.2 vs. 60.0%, *p* < 0.001). Multivariable regression analysis showed that age (OR 0.961; 95% CI 0.940–0.982) and current smoking (OR 1.978; 95% CI 1.145–3.417) were associated with ACS. While 55 drivers (85%) who remained conscious after ACS could seek medical attention without causing accidents, the other 10 (15%) who sustained cardiac arrest caused accidents.

**Conclusions:**

The association between current smoking and ACS occurring while driving suggests that smoking cessation is advised for smokers who drive from the standpoint of driving safety. We expect that prospective studies be conducted to verify our findings and identify individuals at risk for ACS while driving.

**Electronic supplementary material:**

The online version of this article (10.1186/s12199-017-0689-5) contains supplementary material, which is available to authorized users.

## Background

Acute coronary syndrome (ACS) may occur during any human activity, including driving [1–3]. The objectives of this study were to report the frequency and patient characteristics of ACS occurring while driving. An effort was made to identify variables associated with ACS occurring while driving. Furthermore, the behavioral patterns of afflicted drivers were analyzed to clarify relationship between ACS and automobile accidents.

## Methods

This was a single-center, retrospective observational study conducted using prospectively acquired data. The study protocol was approved by our institution’s ethics committee. ACS was defined as the presence of either ST-segment elevation myocardial infarction (STEMI), non-ST-segment elevation myocardial infarction, or unstable angina pectoris [4]. ACS was generally treated according to the recent guidelines [5]. After arrival in the emergency department (ED), the temporal sequence of resuscitative events was recorded on an integrated clinical database CAP-2000 (Nihon Kohden, Tokyo, Japan) by ED residents. Detailed information on when, where, and how the patients’ symptoms developed was obtained from patients, surrogates, or paramedics. They were also asked about activities performed immediately before symptom onset. We modified the classification of daily activities by Hayashi et al., in which the activities were classified into eight categories (sleeping, resting, eating, walking, hard working, bathing/toilet, driving, and other activities) [6, 7].

Unless contraindicated, patients suspected of ACS routinely underwent contrast-enhanced whole-body computed tomography to rule out aortic dissection. Patients diagnosed with ACS were brought to a catheter lab for possible percutaneous coronary intervention (PCI). We used a dataset of 1605 newly diagnosed ACS patients, aged ≥ 18 years, who were admitted to our institution between January 2011 and December 2016. Patients who drove to our institution with symptoms that had occurred while performing other activities as well as those with stable angina pectoris who noticed chest pain while driving were excluded from analysis. Geriatric patients who were transferred from nursing care facilities were also excluded.

### Statistical analysis

Fisher’s exact test was used to compare differences in categorical variables, and Student’s *t* test was used to compare differences in numerical variables. Numerical data are expressed as the mean ± SD, and *p* < 0.05 was considered statistically significant. Multivariable logistic regression analysis was performed using JMP software (SAS Institute, Cary, NC, USA) to identify variables associated with ACS occurring while driving [8]. The variables included age, sex, proportion of STEMI, and cardiovascular risk factors (hypertension, past history of ischemic heart diseases, dyslipidemia, diabetes mellitus, and current smoking). Drinking was not included in the risk factors because making a distinction between habitual and social drinkers was difficult on our database.

## Results

### Frequency

The 1605 ACS patients consisted of 1272 men and 333 women with mean age of 65.8 ± 13.0 years. Among these, 65 (60 men/5 women) sustained ACS while driving. The activities at the time of symptom onset in the other 1540 patients were as follows: sleeping, 284; resting, 361; eating, 89; walking, 227; hard working, 230; bathing/toilet, 91; and other activities, 347. As a result, the frequency of driving among all daily activities at the time of symptom onset was 4.0%. There were 7 professional drivers (10.8%). While 55 drivers presented with acute chest and/or back pain, the other 10 drivers presented with cardiac arrest.

### Demographics

Demographic variables were compared between the 65 patients who sustained ACS while driving and the 1540 patients who sustained ACS while performing other activities. The patients who sustained ACS while driving were significantly younger than those who sustained ACS while performing other activities (57.5 ± 12.2 vs. 66.2 ± 13.0 years, *p* < 0.001) (Table 1). The former group also exhibited significant higher body mass index (BMI) (25.5 ± 5.8 vs. 23.8 ± 3.9 kg/m^2^, *p* < 0.001). Regarding the risk factors, the frequency of current smoking was significantly higher in the former (60.0 vs. 34.2%, *p* < 0.001). However, the frequencies of other risk factors did not differ significantly.

**Table 1:** Comparison of variables between patients who sustained ACS while driving and those who sustained ACS while performing other activities

	Driving (*n* = 65) vs. other activities (*n* = 1540)	
Demographics		*p*
Age (mean ± SD, years)	57.5 ± 12.2 vs. 66.2 ± 13.0	< 0.001*
Male:female	60:5 vs. 1212:328	0.007*
Body mass index (kg/m^2^)	25.5 ± 5.8 vs. 23.8 ± 3.9	< 0.001*
STEMI	34 (52.3%) vs. 701 (45.5%)	0.128
Risk factors		
Hypertension	36 (55.4%) vs. 854 (55.5%)	1.000
Ischemic heart diseases	18 (27.7%) vs. 489 (31.8%)	0.783
Dyslipidemia	23 (35.4%) vs. 461 (29.9%)	0.338
Diabetes mellitus	17 (26.2%) vs. 467 (30.3%)	0.581
Current smoking	39 (60.0%) vs. 526 (34.2%)	< 0.001*

*STEMI* ST-segment elevation myocardial infarction

*Statistically significant

Regarding the relationship between gender and smoking status, the male group showed significantly higher frequency of current smoking than the female counterpart (520/1272 vs. 46/333, *p* < 0.001). Subsequently, demographics including smoking status were compared between 60 male patients who sustained ACS while driving and 1212 male patients who sustained ACS while doing other activities (Additional file 1: Table S1). While the frequency of current smoking was significantly higher in the former (63.3 vs. 39.7%, *p* < 0.001), there were no significant intergroup differences in other demographics.

### Multivariable logistic regression analysis

Multivariable regression analysis was conducted to identify variables associated with ACS occurring while driving, and the results are summarized in Table 2. Age (OR 0.961; 95% CI 0.940–0.982; *p* < 0.001) and current smoking (OR 1.978; 95% CI 1.145–3.417; *p* = 0.015) were found to be associated with ACS occurring while driving (Table 2).

**Table 2:** Multivariable logistic regression analysis to identify variables associated with ACS occurring while driving

Variables	OR	95% CI	*p*
Age	0.961	0.940–0.982	< 0.001*
Male sex	1.816	0.703–4.694	0.218
Hypertension	0.723	0.417–1.254	0.249
Ischemic heart diseases	0.801	0.439–1.463	0.471
Dyslipidemia	0.748	0.430–1.301	0.304
Diabetes mellitus	1.080	0.596–1.959	0.799
Current smoking	1.978	1.145–3.417	0.015*

*Statistically significant

### Response of drivers to ACS

**Fig. 1:**
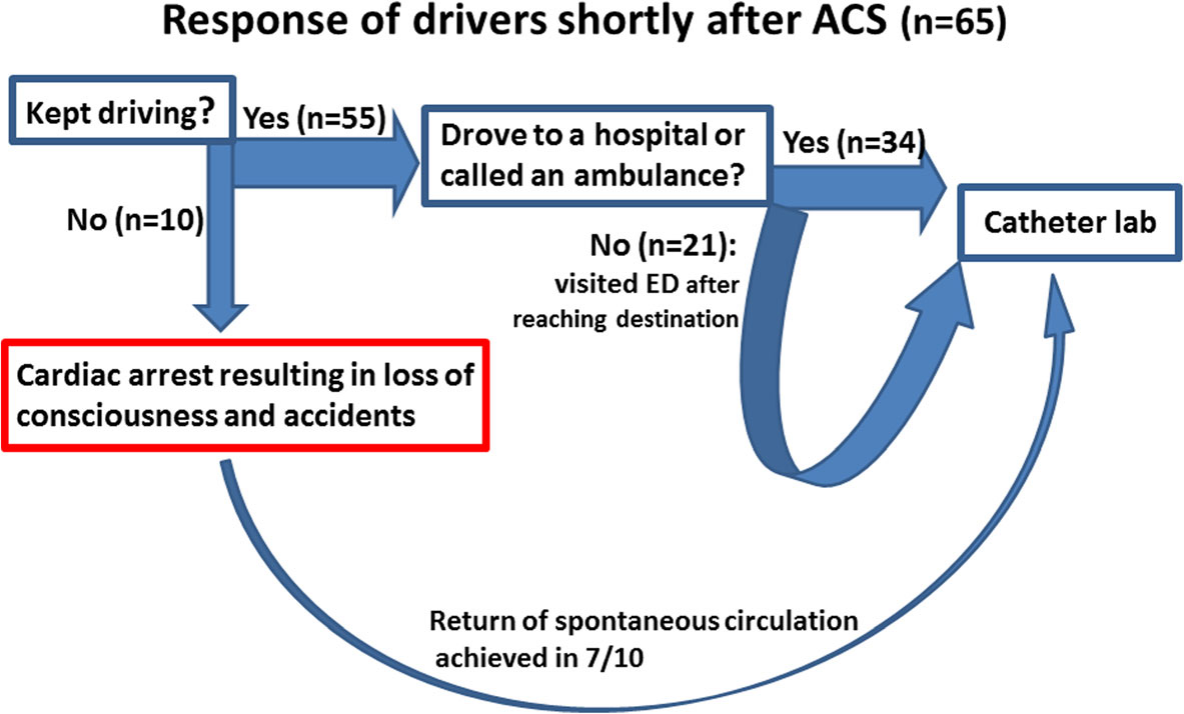
The response of 65 drivers to ACS is illustrated. Fifty-five (85%) kept driving. Among them, 34 either drove directly to a local hospital or called an ambulance for help. The other 21 drivers visited the emergency room (ER) after reaching their destinations. The remaining 10 drivers (15%) sustained cardiac arrest with subsequent loss of consciousness and automobile accidents. Seven of 10 drivers with cardiac arrest achieved return of spontaneous circulation after resuscitation and were taken to the catheter lab

The response of the 65 drivers shortly after ACS is illustrated in Fig. 1. While 10 drivers became comatose and were unable to keep driving because of cardiac arrest, the other 55 (85%) managed to keep driving after sustaining ACS. Among these, 34 patients (62%) could not complete their intended activities because of symptoms; more precisely, they either drove directly to a local hospital or pulled over and called an ambulance for help. The other 21 (38%) could complete their intended activities, i.e., driving to their destinations, and sought medical attention afterwards. Of the 1540 patients who sustained ACS while doing other activities, 1248 were conscious at the time of onset. While 655 patients (52%) could not complete their intended activities because of symptoms, the other 593 (48%) could complete their intended activities and sought medical attention afterwards. Regarding the behavioral patterns, i.e., whether patients’ activities were interrupted by symptoms or not, there was no significant intergroup difference (*p* = 0.21).

None of the 55 drivers who remained conscious after ACS caused automobile accidents. The other 10 drivers (15%) caused automobile accidents after sustaining ACS-induced cardiac arrest. Seven of 10 patients with cardiac arrest achieved return of spontaneous circulation after resuscitation and underwent PCI (Fig. 1).

### Circadian variation and ACS occurring while driving

**Fig. 2:**
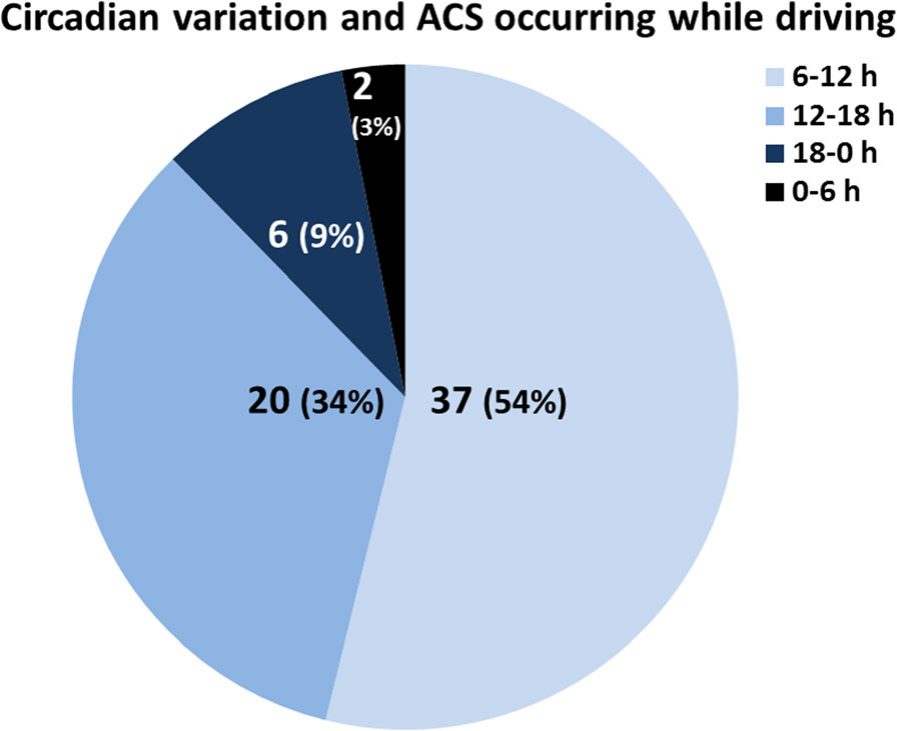
Twenty-four-hour day was divided into four time zones (6–12, 12–18, 18–0, and 0–6 h) and whether ACS while driving was more likely to occur in the specific time zone investigated. Fifty-four percent of driving-related ACS had occurred in the morning

We divided a 24-h day into four time zones (6–12, 12–18, 18–0, and 0–6 h) and investigated whether ACS was more likely to occur in a specific time zone. The number of drivers who sustained ACS in each quartile was 37, 20, 6, and 2, respectively (Fig. 2). Fifty-four percent of ACS while driving had occurred in the first quartile.

## Discussion

Acute cardiovascular events occurring while driving have been studied relatively frequently [1–3]. However, most of those studies were conducted by either forensic scientists or crash scene investigators, and only drivers whose cardiovascular events led to loss of consciousness and subsequent automobile accidents have been evaluated [1–3]. In this context, the present study was unique as it focused on all patients who sustained ACS while driving. The frequency of ACS occurring while driving in our cohort, consisting of adults who led independent lives, was 4.0%. There was a marked circadian variation in the frequency of ACS occurring while driving, with more than 50% of drivers experiencing symptoms in the morning (Fig. 2). While this may have been simply because traffic volume was heavy with commuters in the morning, it may have also reflected the fact that ACS in itself is most likely to occur in the morning [9].

It is likely that ACS occurred while driving merely by chance in the great majority of patients. In other words, causality between driving and ACS is mostly questionable. Nevertheless, demographic comparison (Table 1) as well as multivariable regression analysis (Table 2) suggests that current smoking may be a risk factor for ACS occurring while driving. Smoking promotes hypercoagulability via the action of carbon monoxide [10]. In addition, drivers sit in a confined space in the same posture for hours, and uninterrupted sitting while driving per se may be associated with hypercoagulability [11–14]. Furthermore, drivers may become dehydrated after driving for hours without drinking water. Higher BMI in the afflicted drivers (Table 1) also suggests that underlying obesity may have some role in the causation of ACS occurring while driving. Taken together, combination of these adverse factors may result in thrombosis of the coronary arteries and ACS. In addition to current smoking, age was correlated with ACS: younger adult drivers might be more likely to sustain ACS than their elderly counterparts (Table 2). However, interpretation of this finding requires caution, because driving, smoking habit, and aging process is complicatedly intertwined: the elderly generally drives less frequently and for shorter distances than the younger population, and the smoking rate drops as age advances [15, 16]. Male sex was not associated with ACS (Table 2), and it remains unclear whether gender difference influences the likelihood of developing ACS while driving. Nevertheless, male smokers may be at increased risk for ACS while driving (Additional file 1: Table S1). The fact that women drive less often and cease driving earlier than men should also be considered in interpreting the marked male preponderance of 92% (Table 1) [17]. Based on these findings, smoking cessation is advised for drivers who smoke from a standpoint of driving safety. A recent study showing the efficacy of smoking ban in the reduction of ACS suggests that regulations to limit the use of cigarette while driving may be worth consideration [18].

Regarding the relationship between ACS and automobile accidents, the majority of drivers who sustained ACS kept driving without accidents and managed to reach medical facilities (Fig. 1). By contrast, all 10 patients who sustained cardiac arrest while driving caused automobile accidents. It is likely that loss of consciousness after cardiac arrest resulted in loss of control of vehicles and accidents. We expect that devices to enable earlier detection of driver deterioration are installed in future vehicles to prevent or reduce automobile accidents.

There are limitations to the present study. First, sample number was relatively small due to the single-center retrospective study design. Moreover, this was not a population-based study, and the incidence of ACS occurring while driving remains unknown. Second, the information on the temporal relationship between driving and onset of symptoms, mostly acute chest pain, was mostly obtained from the patients themselves, indicating the possibility of a recall bias. Finally, despite the apparent adverse effects of smoking, information on the quantity of cigarettes consumed by drivers was not always collectible. It was also unclear how many drivers sustained ACS while they were smoking in their vehicles. Despite these limitations, the present study is probably the first to document the frequency and patient characteristics of ACS occurring while driving. As the elderly population grows rapidly, the incidence of ACS occurring while driving may also increase worldwide. Prospective multicenter studies should be conducted to identify individuals at risk for ACS while driving.

## Additional file

Additional file 1: Table S1. Comparison of variables between male patients who sustained ACS while driving and male patients who sustained ACS while performing other activities. (DOC 32 kb)

## Abbreviations

ACS: Acute coronary syndrome
BMI: Body mass index
CI: Confidence interval
ED: Emergency department
OR: Odds ratio
PCI: Percutaneous coronary intervention
SD: Standard deviation
STEMI: ST-segment elevation myocardial infarction
