# Supplementary material for: Acute coronary syndromes occurring while driving: frequency and patient characteristics
Source: Environ Health Prev Med. 2017 Dec 20;22:82. doi: 10.1186/s12199-017-0689-5 (PMC5738902; doi:10.1186/s12199-017-0689-5)
Supplement: Additional file 1: Table S1. — Comparison of variables between male patients who sustained ACS while driving and male patients who sustained ACS while performing other activities. (DOC 32 kb) [file 12199_2017_689_MOESM1_ESM.doc]

**Supplementary File** Comparison of variables between male patients who sustained ACS while driving and male patients who sustained ACS while performing other activities

|  | **Driving (n=60) vs. Other activities (n=1212)** |  |
| --- | --- | --- |
| **Demographics** |  | ***p*** |
| **Age (mean ± SD, yrs)** | 57.0 ± 12.4 vs. 64.5 ± 12.8 | < 0.001* |
| **Risk factors** |  |  |
| Hypertension  Ischemic heart diseases  Dyslipidemia  Diabetes mellitus  Current smoking | 33 (55.0%) vs. 645 (53.2%)  15 (25.0%) vs. 390 (32.2%)  20 (33.3%) vs. 354 (29.2%)  15 (25.0%) vs. 368 (30.3%)  38 (63.3%) vs. 526 (39.7%) | 0.894  0.260  0.472  0.471  < 0.001** |

*, **: statistically significant
